# Effects of Poultry Manure on the Growth, Physiology, Yield, and Yield-Related Traits of Maize Varieties

**DOI:** 10.1021/acsomega.3c00880

**Published:** 2023-06-01

**Authors:** Ayesha Rasool, Abdul Ghani, Rab Nawaz, Saliha Ahmad, Khurram Shahzad, Ansa Rebi, Baber Ali, Jinxing Zhou, Muhammad Ibrar Ahmad, Muhammad Faran Tahir, Mona S. Alwahibi, Mohamed S. Elshikh, Sezai Ercisli

**Affiliations:** † Department of Botany, University of Sargodha, Sargodha 40100, Pakistan; ‡ Department of Biology, Case Western Reserve University, Cleveland, Ohio 44106-7078, United States; § Jianshui Research Station, School of Soil and Water Conservation, Beijing Forestry University, Beijing 100083, China; ∥ Department of Plant Sciences, Quaid-i-Azam University, Islamabad 45320, Pakistan; ⊥ Soil and Water Testing Laboratory, Sargodha 40100, Pakistan; # Department of Plant Pathology, University of Agriculture, Faisalabad 38000, Pakistan; ∇ Department of Botany and Microbiology, College of Science, King Saud University, Riyadh 11451, Saudi Arabia; ○ Department of Horticulture, Agricultural Faculty, Ataturk Universitesi, Erzurum 25240, Türkiye; ◆ HGF Agro, Ata Teknokent, Erzurum 25240, Türkiye

## Abstract

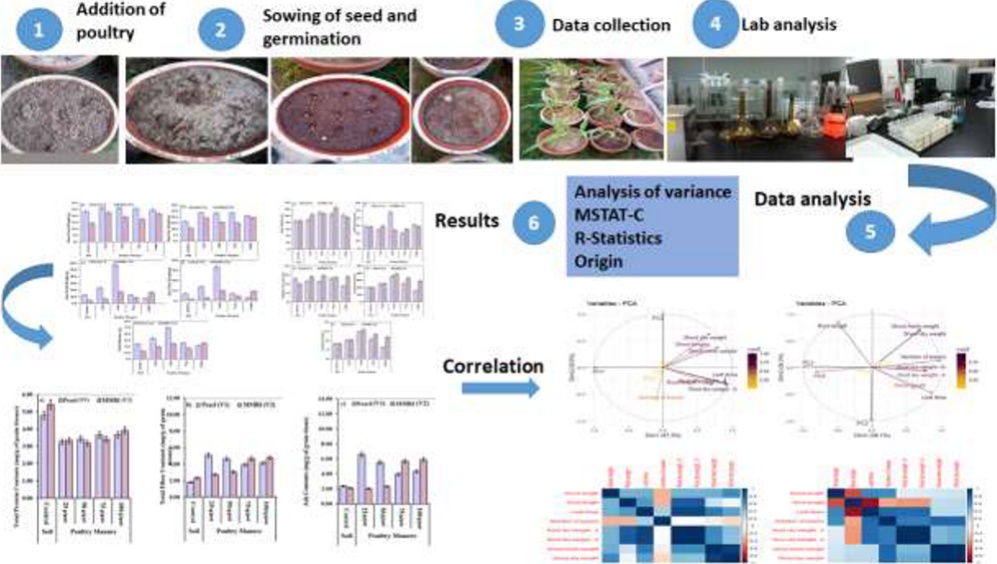

Industries play a significant role in the improvement of lifestyle and in the development of a country. However, the byproducts from these industries are a source of environmental pollution. The proper use of the byproducts of these industries can help to cope with environmental pollution. Some byproducts have high nutritional content and are good for crop plants. The purpose of this research was to investigate the effect of different rates of poultry manure on the soil chemical properties, growth, and yield of maize. A pot experiment was conducted in the botanical garden of the Department of Botany, University of Sargodha, Pakistan to investigate the effect of various treatments of poultry manure (0, 25, 50, 75, and 100 g/pot) on the morphological, physiological, and yield attributes of two maize varieties, Pearl and MMRI. Treatment T_1_ was a mixture of soil and 75 g/pot poultry manure, T_2_ was a mixture of soil and 50 g/pot poultry manure, T_3_ was a mixture of soil and 25 g/pot poultry manure, and T_4_ was 100 g/pot poultry manure. Soil without any industrial byproduct (100% soil only) was used as the control (T_0_). The results revealed that the use of poultry manure enhanced the physical properties of the soil. Available P and soil organic matter were improved in soil amended with poultry manure. It is evident from the results that the vegetative growth of both maize varieties was significantly enhanced by growing in soil amended with poultry manure as compared to their respective control. Similar responses were also recorded for the physiological attributes of leaf area, photosynthetic rate, transpiration rate, stomatal conductance, and water use efficiency of both varieties. Yield and yield-contributing traits of both maize varieties were significantly improved by growing plants in soil amended with 50 and 75 g/pot of poultry manure. It is also inferred that the use of 50 g/pot poultry manure in soil amendment is an eco-friendly and economically effective option for maize growers of arid and semiarid regions to enhance the kernel yield and profit per annum. Poultry manure could be useful to ameliorate the adverse effects of salinity stress on all parameters, particularly the grain yield. Furthermore, this would be a useful and economical method for the safe disposal of byproducts.

## 1 Introduction

Climate is one of the vital factors influencing soil-forming processes and properties.^1^ Although the global climate has been constantly changing throughout geological earth history, the extent to which current changes occur at the human life scale is dramatic.^2^ The global average temperature is estimated to increase by another 2–3 °C by the end of 21st century.^3^ However, the impact of these changes on soil is not predictably directional, resulting in changes that may vary in strength, occurrence, and outcome. Increasing level of atmospheric CO_2_ concentration, temperature, drought stress, uneven precipitation, and atmospheric N_2_ deposition in the soil have drastic impacts on soil texture and soil nutrients. Therefore, agricultural productivity largely depends on the efficient use of soil nutrients and organic amendments. In recent times, poultry manure (PM) has gained attention as a potential source of organic fertilizer due to its high nutrient content and relatively low cost. PM, which is rich in nitrogen, phosphorus, potassium, and other essential nutrients, has been shown to improve soil fertility, increase crop yield, and enhance the quality of agricultural products.^4^ Nitrogen (N) is the major growth hampering mineral nutrient for agricultural crops across the globe.^5^ Moreover, there is an evidence that crop yields have significantly decreased due to improper and less availability of nutrients with increasing aridity under changing climate.^6−8^ Worldwide reduction in cultivable land by urbanization and industrialization is also leading to a food crisis.^9^ Food and Agriculture Organization estimated that by 2050, feeding a world population of 9.1 billion would require approximately 70% more food than available at present.^10^ Thus, for ensuring food security, there is a dire need for advanced technologies, modern cultural practices, and more productive cultivars.^11,12^ Under such a scenario, nitrogen-containing organic substances could be utilized as an effective and economic alternative to expensive synthetic N fertilizers, with a documented potential to improve crop yields and soil properties.^13,14^ We assume that N application as organic manures may nullify the low availability of N in soil and its uptake in maize.^15^ Thus, this study was executed to explore the role of PM from the poultry industry as a nitrogen source in improving maize growth and yield with improved nutritional contents.

Maize (*Zea mays*), also well known as corn, is an important staple cereal crop worldwide belonging to the Poaceae family. It is a nutrient-demanding crop, and therefore adequate and balanced nutrient supply is important in its growth and production.^16^ The use of chemical fertilizers has been reported to increase crop yields, but their use is limited by the high cost, scarcity during the time of its need (planting season), soil acidity, and nutrient imbalance. Because of these, the use of organic manure like PM was found useful in increasing crop production.^17^ PM is cheap, readily available at all times, environmentally friendly, and also has a residual effect and ability to improve soil structure compared with chemical fertilizers.^18^ PM application increases soil N by more than 53%, while exchangeable cations are also increased significantly upon application.^19^ The rate of PM applied may also influence the amount of nutrient released (soil chemical properties), growth, and yield of maize. PM is a useful source of N for maize for its growth and yield.^19^ Therefore, to cater this problem, there is dire need to improve the nitrogen level in the soil for enhanced growth and production of maize. Timsina^20^ reported that using indigenous available organic nutrient source can enhance the efficiency and reduce the quantity of chemical fertilizer required. Apart from enhancing nutrient use efficiency, integrated nutrient use also maintains soil health, enhances yield, and reduces cost of production.

For integrated nutrient management in maize cultivation, PM is usually applied to the prepared soil 2 weeks before planting maize to allow the mineralization of the PM.^21^ Delaying or early application of PM to maize plants may have an implication on the soil chemical properties, growth, and yield of the crop. Many researchers have suggested that N should be applied at the time it is needed by the crop.^22^ During integrated nutrient management involving PM for maize, it is, therefore, necessary to investigate the best time during the growth of the crop to apply PM that will optimize the soil chemical properties, growth, and yield of maize. Overall, this study highlights the importance of optimizing PM application rates to achieve maximum maize yield and quality while minimizing potential negative impacts on soil health and environment. Therefore, the objectives of this study were to investigate the effect of different rates of PM on the soil chemical properties, growth, and yield of maize. The results provide valuable information to farmers, extension workers, and policymakers on the sustainable use of PM as an organic fertilizer for maize production.

## 2 Materials and Methods

### 2.1 Plant Material

Seeds of commercially grown four maize varieties (V_1_: Pearl, V_2_: MMRI yellow, V_3_: Akbar, and V_4_: Sunehri) were obtained from Maize and Millet Institute Yousafwala, Sahiwal, Pakistan. Selected four varieties were evaluated in the pilot experiment at the seed germination stage. Ten seeds of each variety were placed in petri dishes on a filter paper soaked with 10 mL of distilled water. Seeds were germinated for 7 days in complete darkness. Seed germination percentage was observed for all maize varieties, and two varieties having the highest and lowest seed germination rates were selected for further experimentation.

A pot experiment was carried out at the research garden of the Department of Botany, University of Sargodha, Pakistan to evaluate the effect of various treatments of PM on the physico-chemical properties of soil and growth, yield, and nutrient status of the selected maize varieties. Total of five levels (T_0_–T_4_) of PM were used in the experiment using completely randomized design with three replicates of each treatment and variety (Table 1). Treatment T_1_ was a mixture of soil and 75 g/pot PM, T_2_ was a mixture of soil and 50 g/pot PM, T_3_ was a mixture of soil and 25 g/pot PM, and T_4_ was 100 g/pot pure PM. Soil without any industrial byproduct (100% soil only) was used as the control (T_0_). PM was incorporated within soil with different proportions, and soil cover was made so that chances of nutrient losses from soil remain minimum.

**Table 1:** Amendments of Soil with Different Industrial Byproducts for Growth of Maize

sr.	symbol used	soil amendments
1	T_0_	100% soil (control)
2	T_1_	75 g/pot PM
3	T_2_	50 g/pot PM
4	T_3_	25 g/pot PM
5	T_4_	100 g/pot PM

Soil amended with five levels of PM was filled in the pots that were watered for 7 days. After 7 days, the soil samples were subjected to soil analysis to compare the effects of PM levels on the physico-chemical properties of the soil.

### 2.2 Soil Analysis

Soil samples (0–15 cm deep) were taken from three pots for each level of PM. The samples were bulked and air-dried for analysis. Waste material of poultry was used as PM. Organic matter content was determined by the Walkley–Black dichromate digestion method.^23^ Total soil nitrogen was determined by the Kjeldahl method.^24^ Available P was determined by the Bray-1 method, and color was developed in soil extracts using the ascorbic acid blue color method.^25^ Exchangeable K, Ca, and Mg were extracted using ammonium acetate. K was determined on a flame photometer and Ca and Mg were determined by EDTA titration. The soil pH in 0.01 M CaCl_2_ was determined using a glass electrode (Table 2).

**Table 2:** Physico-Chemical Properties of Soil Amended with Different Industrial Byproducts

sr.	symbol	pH	EC	phosphorus	potassium	saturation	soil texture
1	T_0_	8.4	1.8	11.9	1.22	46	clay loam
2	T_1_	6.6	7.52	97.1	425	44	loam
3	T_2_	6.9	7.22	66.5	390	40	loam
4	T_3_	7.2	5.64	15.4	450	40	loam
5	T_4_	6.7	17.3	106.3	600		loam

### 2.3 Seed Sowing and Growth Conditions

Seeds of the two selected varieties were surface-sterilized by treating them with mercuric chloride to remove various biotic and abiotic agents. The selected seeds were soaked in 0.1% solution of HgCl_2_ for 5 min and were washed with distilled water to remove the traces of mercuric chloride. After the surface sterilization, the seeds were soaked in distilled water for 20 min and were sown in pots at a standard depth of 2 cm. Plant populations were thinned to four plants per pot after 7 days of seedling emergence. All plants were grown for 120 days in pots filled with different mixtures of soil and PM. Samples were collected at the vegetative and maturity stages for evaluation of PM treatments on the growth, yield, and nutrient status of both maize varieties.

### 2.4 Growth Parameters

Plants were harvested at the vegetative stage (30 days after sowing) and at maturity (65 days after sowing). Harvested plants were washed with tap water to remove the soil particles. Data were recorded for different morphological attributes (plant height, root length, number of leaves, leaf area, leaf area index, shoot fresh weight, shoot dry weight, root fresh weight, root dry weight, and total biomass production). All measurements were recorded per plant from each replicate and treatment of both maize varieties. Plant height was measured from the base to the tip of stem with a measuring tape from each replicate, and the average plant height was expressed in centimeter (cm). Root length was measured from the base to the root tip of each plant with a measuring tape in centimeter (cm). Total number of leaves per plant were counted from three replicates of each treatment. A leaf area meter was used to measure the leaf area per plant in centimeter square (cm^2^), and leaf area index was calculated by dividing the leaf area by the land area covered by the plant. Fresh weight of shoot was measured with the help of an electronic balance, and the average fresh weight was expressed in grams (g). Fresh weight of root was measured with the help of an electronic balance, and the average fresh weight was expressed in grams (g). After recording of fresh weight, same shoots were dried in an oven at 70 °C for 3 days, and dry weight was recorded using an electronic balance. After recording of fresh weight, same roots were dried in an oven at 70 °C for 3 days, and dry weight was recorded using an electronic balance. Total plant biomass was also measured using an electronic balance.

### 2.5 Physiological Attributes

Data for physiological attributes were collected from the intact leaves of plants at vegetative (30 days after sowing) and at maturity (65 days after sowing) stages. A fully expanded leaf (the second leaf from top) of each plant was selected for all physiological measurements. The selected leaf was placed in a chamber of portable infrared gas analyzer from 10.00 a.m. to 02.00 p.m. under bright sunlight. Before recording the data, following adjustments were made on the photosynthesis system of the device. Atmospheric pressure was 97.9 KPa, air per unit leaf area was 403.3 mmol m^–2^ s^–1^, temperature of the leaf ranged from 28.4 to 32.4 °C, ambient temperature ranged from 22.4 to 27.9 °C, ambient CO_2_ concentration was 354 μmol mol^–1^, water vapor pressure into the chamber ranged from 6.0 to 8.9 mbar, and PAR at the leaf surface was maximum upto 1711 mol m^–2^ s^–1^. Average values of photosynthetic rate, transpiration rate, stomatal conductance, and water use efficiency (WUE) were expressed in μmol m^–2^ s^–1^, mmol m^–2^ s^–1^, mmol m^–2^ s^–1^, and pmol CO_2_ mmol^1^ H_2_O, respectively. Measurements for photosynthesis rate in terms of net CO_2_ assimilation rate (*Pn*), transpiration rate (*E*), stomatal conductance to water (*g*_s_), leaf temperature, and humidity were recorded, and WUE was calculated by the following equation.
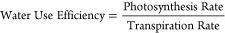


### 2.6 Yield and Yield Components

Agronomic data regarding the final yield and yield-related components were collected from each replicate and treatment of both maize varieties after harvesting the crop. Cob length was measured in centimeters using a measuring tape for the plant growing in each treatment. Cob diameter was measured in centimeter squares with a digital vernier calipers for the plant growing in each treatment. Cob weight was measured in grams using an electronic balance for the plant growing in each treatment. Number of grains per cob were counted for each plant grown in each treatment. Biological yield was measured per plant basis in grams for each variety grown in each treatment.

Grain yield was measured per plant basis in grams for each variety grown in each treatment. Harvest index was computed by using the formula given by Donald and Hamblin^26^ for each genotype and was expressed in %.
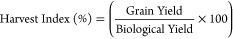


### 2.7 Statistical Analysis

Data collected at different crop stages were analyzed statistically using the analysis of variance (ANOVA) technique, and the MSTAT-C computer program was used for this purpose. The least significant difference test at 5% probability level was used to assess the differences among significant means.^27^

## 3 Results

### 3.1 Morphological Traits

#### 3.1.1 Plant Height (cm)

**Figure 1:**
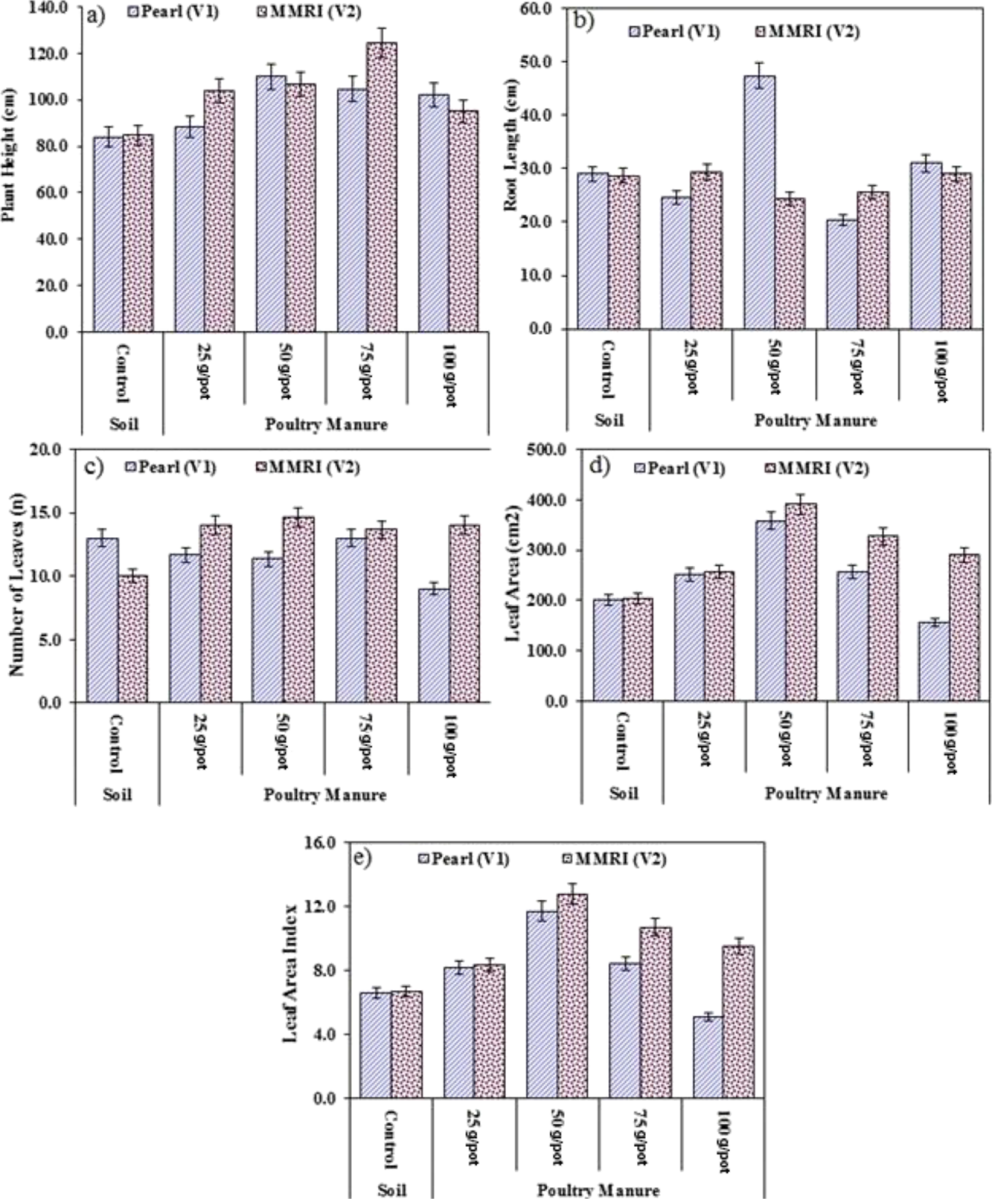
Effect of PM on the (a) plant height (cm), (b) root length (cm), (c) number of leaves (n), (d) leaf area (cm^2^), and (e) leaf area index.

ANOVA presented in Table 6 depicts the significant variation between maize varieties and PM treatments and their combined interaction of varieties on the plant height. A significant increase in the plant height of Pearl (ranging from 5.1 to 31.0%) and MMRI (ranging from 12.1 to 46.9%) was observed as compared to their respective control in response to various doses of PM (25, 50, 75, and 100 g/pot) (Table 3). In Pearl (V_1_), minimum increase (88.3 cm) in the plant height was obtained at 25% of PM and maximum (110 cm) at 50 g/pot of PM, whereas in the case of MMRI (V_2_), minimum increase (95.0 cm) was observed at 100 g/pot of PM and maximum (124.5 cm) at 75 g/pot of PM in the soil as compared to control (Figure 1).

**Table 3:** Effect of PM on Growth-Related Traits of Maize Varieties^a^

treatments	V_1_ V_2_		V_1_ V_2_		V_1_ V_2_		V_1_ V_2_		V_1_ V_2_	
	plant height (cm)		root length (cm)		no of leaves (*n*)		leaf area (cm^2^)		leaf area index	
T_0_	84.0	84.7	29.0	28.7	13.0	10.0	201.0	203.7	6.6	6.7
T_1_	88	103	24.7	29.3	11.7	14.0	251.0	255.7	8.2	8.4
T_2_	110	106	47.3	24.3	11.3	14.7	358.0	390.7	11.7	12.8
T_3_	104	124	20.3	25.5	13.0	13.7	257.0	327.0	8.4	10.7
T_4_	102	95	31.0	29.0	9.00	14.0	155.3	290.7	5.1	9.5

a V_1_ represents pearl, and V_2_ represents MMRI in the table.

#### 3.1.2 Root Length (cm)

ANOVA presented in Table 6 depicts the significant variation between maize varieties and PM treatments and their combined interaction of varieties on the root length. A significant increase in the root length of Pearl (63.2 and 6.9%) was measured at 50 and 100 g/pot of PM, while a decrease of 14.9 and 29.9% was observed in the root length at 25 and 75 g/pot of PM as compared to the control. On the other hand, a negligible increase (2.2 and 1.0%) in the root length was recorded at 25 and 100 g/pot of PM, whereas the root length decreased at 50 and 75 g/pot amendments of soil, that is, 15 and 11%, respectively (Figure 1 and Table 3).

#### 3.1.3 Number of Leaves

ANOVA presented in Table 6 depicts the significant variation between maize varieties and PM treatments and their combined interaction of varieties on the number of leaves per plant. A significant decrease in the number of leaves of Pearl ranged from 10.3 to 30.8% at 25, 50, and 100 g/pot of PM over the control, but the number of leaves (13 leaves) remained constant at 75 g/pot as in the control. Number of leaves were increased in MMRI (ranging from 36.7 to 46.7%) as compared to their respective control. In Pearl (V_1_), minimum number of leaves (9.0 leaves) were obtained at 100 g/pot of PM and maximum (13 leaves) at 75 g/pot of PM, whereas in the case of MMRI (V_2_), minimum number of leaves (13.7) were observed at 75 g/pot of PM and maximum (14.7 leaves) at 50 g/pot of PM in the soil as compared to the control (Figure 1 and Table 3).

#### 3.1.4 Leaf Area (cm^2^)

ANOVA presented in Table 6 depicts the significant variation between maize varieties and PM treatments and their combined interaction of varieties on the leaf area per plant. A significant increase of 24.9, 78.1, and 27.9% in leaf area (ranging from 251.0 to 358.0 cm^2^) of Pearl was observed at 25, 50, and 75 g/pot of PM over control, but the leaf area decreased up to 22.7% with an average of 155.3 cm^2^ at 100 g/pot of PM as compared to the control. Leaf area was increased in MMRI (ranging from 25.5 to 91.8%) as compared to their respective control. Minimum leaf area (255.7 cm^2^) was observed at 25 g/pot of PM and maximum (390.7 cm^2^) at 50 g/pot of PM in the soil as compared to the control (Figure 1 and Table 3).

#### 3.1.5 Leaf Area Index (cm^2^)

ANOVA presented in Table 6 depicts the significant variation between maize varieties and PM treatments and their combined interaction of varieties on the leaf area index per plant. A significant increase of 24.7, 77.8, and 27.7% in the leaf area index (ranging from 8.2 to 11.7) of Pearl was observed at 25, 50, and 75 g/pot of PM over control, but the leaf area index decreased up to 22.8% with an average of 5.1 at 100 g/pot of PM as compared to the control. Leaf area index was increased in MMRI (ranging from 25.1 to 91.2%) as compared to their respective control. Minimum leaf area index (8.4) was observed at 25 g/pot of PM and maximum (12.8) at 50 g/pot of PM in the soil as compared to the control (Figure 1 and Table 3).

#### 3.1.6 Shoot Fresh Weight (g)

**Figure 2:**
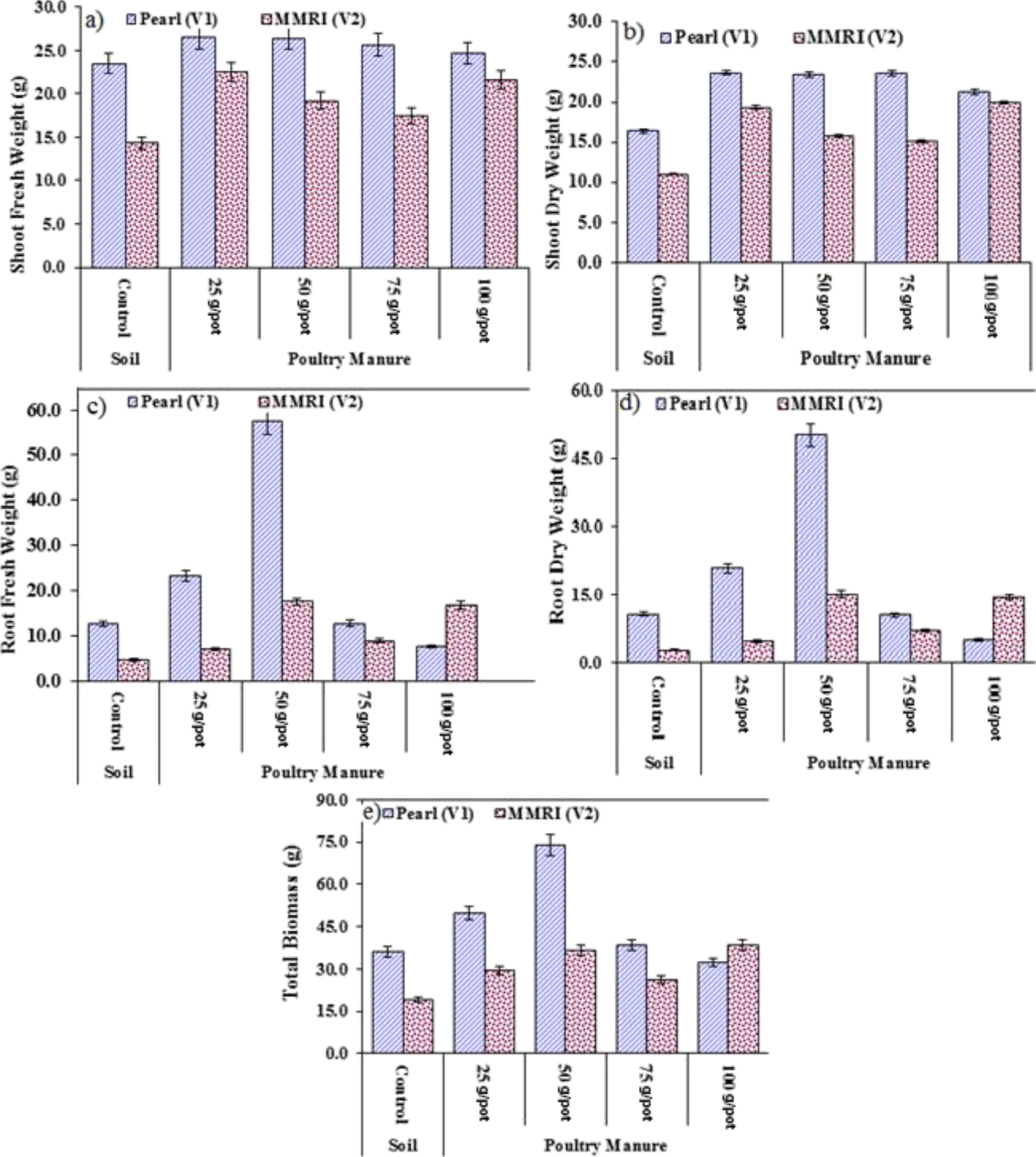
Effect of PM on the (a) shoot fresh weight (g), (b) root fresh weight (g), (c) shoot dry weight (g), (d) root dry weight (g), and (e) total biomass (g).

ANOVA presented in Table 6 depicts the significant variation between maize varieties and PM treatments and their combined interaction of varieties on the shoot fresh weight. A significant increase in the shoot fresh weight of Pearl (ranging from 5.1 to 12.5%) and MMRI (ranging from 22.1 to 57.3%) was observed as compared to their respective control. In Pearl (V_1_), minimum increase (24.7 g) in the shoot fresh weight was obtained at 100 g/pot of PM and maximum (26.4 g) at both 25 and 50 g/pot of PM, whereas in the case of MMRI (V_2_), minimum increase (17.5 g) was observed at 75 g/pot of PM and maximum (22.5 g) at 25 g/pot of PM in the soil as compared to the control (Figure 2 and Table 4).

**Table 4:** Effect of PM on Growth-Related Traits of Maize Varieties^a^

treatments	V_1_ V_2_		V_1_ V_2_		V_1_ V_2_		V_1_ V_2_		V_1_ V_2_	
	shoot fresh weight (g)		shoot dry weight (g)		root fresh weight (g)		root dry weight (g)		total biomass (g)	
T_0_	23.5	14.3	16.4	11.1	12.6	4.6	10.7	2.7	36.1	18.9
T_1_	26.4	22.5	23.6	19.3	23.2	6.9	20.8	4.8	49.7	29.4
T_2_	26.4	19.2	23.5	15.9	57.4	17.5	55.2	15.1	83.8	36.6
T_3_	25.6	17.5	23.5	15.2	12.7	8.8	10.5	7.0	38.3	26.3
T_4_	24.7	21.6	21.3	19.9	7.6	16.7	5.2	14.4	32.3	38.2

a V_1_ represents pearl, and V_2_ represents MMRI in the table.

#### 3.1.7 Shoot Dry Weight (g)

ANOVA presented in Table 6 depicts the significant variation between maize varieties and PM treatments and their combined interaction of varieties on the shoot dry weight. A significant increase in the shoot dry weight of Pearl (ranging from 30.1 to 43.9%) and MMRI (ranging from 36.6 to 79.6%) was observed as compared to their respective control. In Pearl (V_1_), minimum increase (21.3 g) in the shoot dry weight was obtained at 100 g/pot of PM and maximum (∼23 g) at 25, 50, and 75 g/pot of PM. In the case of MMRI (V_2_), minimum increase (15.2 g) was observed at 75 g/pot of PM and maximum (∼19 g) at both 25 and 100 g/pot of PM in the soil as compared to the control (Figure 2 and Table 4).

#### 3.1.8 Root Fresh Weight (g)

ANOVA presented in Table 6 depicts the significant variation between maize varieties and PM treatments and their combined interaction of varieties on the root fresh weight. A significant increase in the shoot fresh weight of Pearl (ranging from 0.9 to 95.6%) and MMRI (ranging from 7.1 to 29.6%) was observed as compared to their respective control. In Pearl (V_1_), minimum increase (12.7 g) in the root fresh weight was obtained at 75 g/pot of PM and maximum (57.4 g) at 50 g/pot of PM. In the case of MMRI (V_2_), minimum increase (6.9 g) was observed at 25 g/pot of PM and maximum (17.5 g) at 50 g/pot of PM in the soil as compared to the control (Figure 2 and Table 4).

#### 3.1.9 Root Dry Weight (g)

ANOVA presented in Table 6 depicts the significant variation between maize varieties and PM treatments and their combined interaction of varieties on the root dry weight. There was a significant increase in the root dry weight of Pearl (ranging from 94 to 99%) at 25 and 50 g/pot of PM, and the root dry weight decreased at 75 and 100 g/pot of PM, that is, 1.4 and 51%, respectively. In the case of MMRI, an increase in the root dry weight (ranging from 59 to 78%) was observed as compared to the respective control. Overall comparison of all treatments shows that 50 g/pot of PM was the most effective level for optimum root dry weight production (Figure 2 and Table 4).

#### 3.1.10 Total Biomass (g)

ANOVA presented in Table 6 depicts the significant variation between maize varieties and PM treatments and their combined interaction of varieties on total biomass production. There was a significant increase in the total biomass of Pearl (ranging from 6.1 to 72.2%) at 25, 50, and 75 g/pot of PM, while the total biomass decreased up to 10.6% at 100 g/pot of PM, that is, 32.3 g. In the case of MMRI, total biomass production was increased (ranging from 39 to 62.4%) at all treatments of PM as compared to their respective control. Overall comparison of all treatments shows that 50 g/pot of PM was the most effective level for optimum total biomass production, that is, 60.21 g (Figure 2 and Table 4).

### 3.2 Physiological Traits

#### 3.2.1 Leaf Temperature (°C)

**Figure 3:**
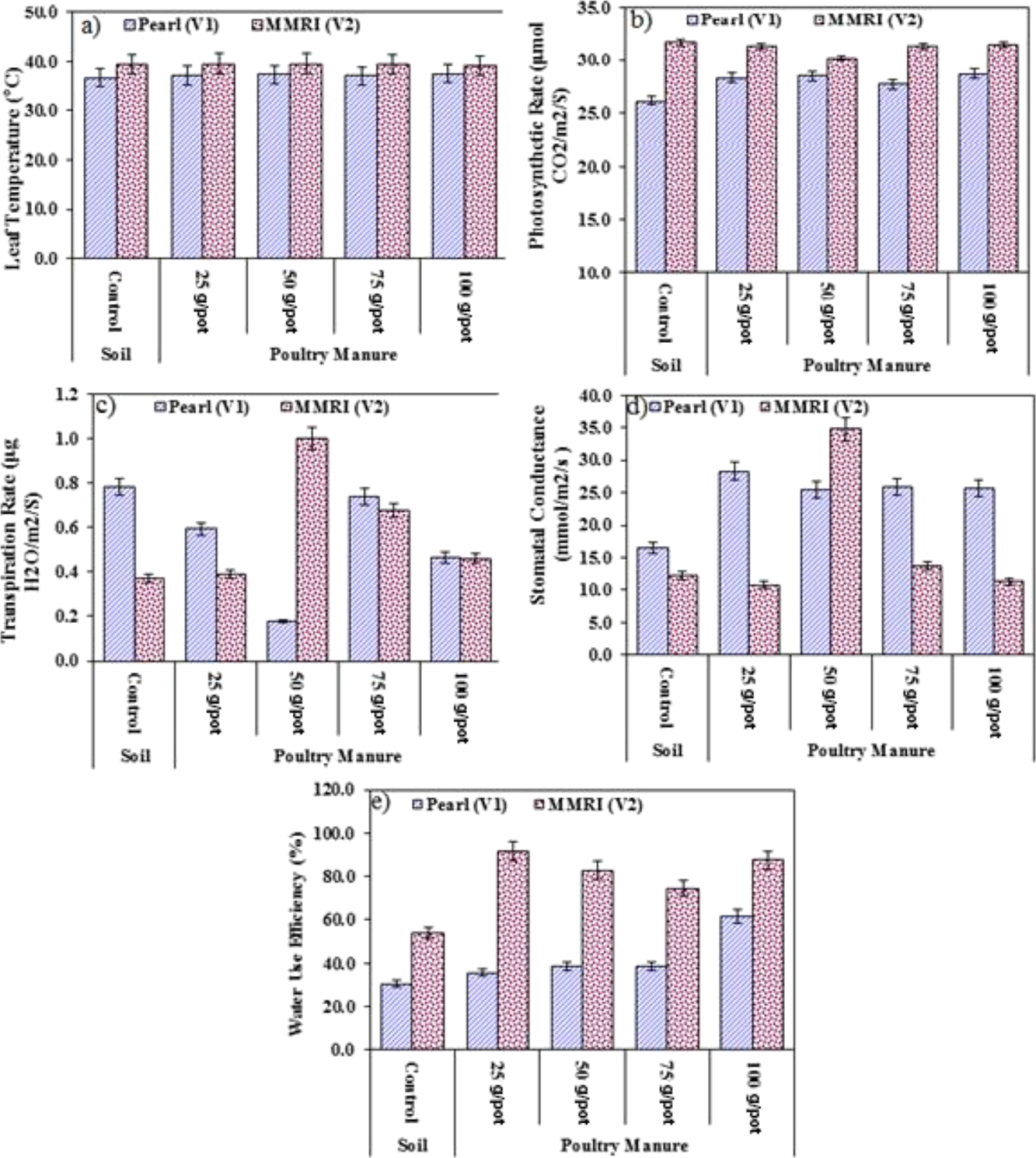
Effect of PM on the (a) leaf temperature (°C), (b) photosynthetic rate, (c) transpiration rate, (d) stomatal conductance, and (e) WUE (%).

ANOVA presented in Table 6 depicts the significant variation between maize varieties and PM treatments and their combined interaction of varieties on the leaf temperature. An insignificant increase ranging from 0.54 and 3.24% in the leaf temperature (ranging from 37.0 to 37.5 °C) of Pearl was observed at various levels of PM over control. Similarly, an increase of 0.17% was observed in the average leaf temperature (39.5 °C) of MMRI grown in soil amended with each treatment of PM (Figure 3 and Table 5).

**Table 5:** Effect of PM on Growth-Related Traits of Maize Varieties^a^

treatments	V_1_ V_2_		V_1_ V_2_		V_1_ V_2_		V_1_ V_2_		V_1_ V_2_	
	leaf temperature (°C)		transpiration rate (μg H_2_O m^–2^ S^–1^)		photosynthetic rate (μmol CO_2_/m^–2^/S^–1^)		stomatal conductance (mmol m^–2^ s^–1^)		WUE (%)	
T_0_	36.6	39.4	0.8	0.4	26.2	31.6	16.5	12.2	30.5	53.8
T_1_	37.1	39.5	0.6	0.4	28.3	31.3	28.2	10.7	35.6	91.6
T_2_	37.3	39.5	0.2	1.1	28.5	30.2	25.4	34.7	38.4	82.7
T_3_	37.0	39.5	0.7	0.7	27.7	31.3	25.9	13.7	38.8	74.6
T_4_	37.5	39.5	0.5	0.5	28.7	31.4	25.5	11.2	61.6	87.4

a V_1_ represents Pearl, and V_2_ represents MMRI.

#### 3.2.2 Photosynthetic Rate (μmol CO_2_ m^–2^ S^–1^)

ANOVA presented in Table 6 depicts the significant variation between maize varieties and PM treatments and their combined interaction of varieties on the photosynthetic rate. A significant increase in the photosynthetic rate of Pearl (ranging from 5.87 to 9.60%) and a decrease in the rate of MMRI (ranging from 0.6 to 4.5%) was observed as compared to their respective control. In Pearl (V_1_), minimum increase (27.7 μmol CO_2_ m^–2^ S^–1^) in the photosynthetic rate was obtained at 75 g/pot of PM and maximum (28.7 μmol CO_2_ m^–2^ S^–1^) at 100 g/pot of PM, whereas in the case of MMRI (V_2_), minimum decrease (31.4 μmol CO_2_ m^–2^ S^–1^) was observed at 100 g/pot of PM and maximum (30.2 μmol CO_2_ m^–2^ S^–1^) at 50 g/pot of PM in the soil as compared to the control (Figure 3 and Table 5).

**Table 6:** Effect of PM on the Different Morphological Attributes of Maize Varieties

source	DF	plant height (cm)	root length (cm)	number of leaves (*n*)	leaf area (cm^2^)	leaf area index
treatment	4	377.7^***^	155.452^***^	6.6709^***^	25091.1^***^	26.9725^***^
variety	1	39.35*	126.926^***^	18.5128^***^	29,116^***^	31.2992^***^
treatment × variety	4	174.35^***^	142.322^***^	12.6795^***^	22007.2^***^	23.6573^***^
error	25	9.63	4.804	3.8077	137.9	0.1483

source	DF	shoot fresh weight (g)	shoot dry weight (g)	root fresh weight (g)	root dry weight (g)	total biomass (g)
treatment	4	76.91^***^	128.01^***^	495.5^***^	479.54^***^	659.5^***^
variety	1	496.04^***^	647.31^***^	1677.36^***^	1577.61^***^	3855.8^***^
treatment × variety	4	74.81^***^	35.11^***^	341.31^***^	342.47^***^	583.2^***^
error	25	0.86	0.84	1.13	1.17	9.5

source	DF	leaf temperature	photosynthetic rate	transpiration rate	stomatal conductance	WUE
treatment	4	1.077^***^	0.37646^***^	0.37646^***^	356.52^***^	1182.26^***^
variety	1	105.235^***^	0.34812^***^	0.34812^***^	0.26^***^	2206.95^***^
treatment × variety	4	2.575^***^	0.30205^***^	0.30205^***^	246.13^***^	1023.05^***^
error	25	0.138	0.00547	0.00547	0.1	3.47

source	DF	cob length (cm)	cob diameter (cm)	cob weight (g)	biological yield (g)	grain yield (g)	harvest index (%)
treatment	4	377.7^***^	162.66^***^	0.093^***^	230.64^***^	2.53^***^	0.024*
variety	1	39.35^***^	86.87^***^	6.034^***^	683.10^***^	5.27*	0.024*
treatment × variety	4	174.35^***^	539.53^***^	0.97^***^	1324.28^***^	16.29^**^	0.014^**^
error	25	9.63	1.96	0.027	1.83	0.05	0.002

source	DF	leaf temperature	photosynthetic rate	transpiration rate	stomatal conductance	WUE
treatment	4	1.077^***^	0.37646^***^	0.37646^***^	356.52^***^	1182.26^***^
variety	1	105.235^***^	0.34812^***^	0.34812^***^	0.26^***^	2206.95^***^
treatment × variety	4	2.575^***^	0.30205^***^	0.30205^***^	246.13^***^	1023.05^***^
error	25	0.138	0.00547	0.00547	0.1	3.47

source	DF	cob length (cm)	cob diameter (cm)	cob weight (g)	biological yield (g)	grain yield (g)	harvest index (%)
treatment	4	377.7^***^	162.66^***^	0.093^***^	230.64^***^	2.53^***^	0.024*
variety	1	39.35^***^	86.87^***^	6.034^***^	683.10^***^	5.27*	0.024*
treatment × variety	4	174.35^***^	539.53^***^	0.97^***^	1324.28^***^	16.29^**^	0.014^**^
error	25	9.63	1.96	0.027	1.83	0.05	0.002

source	DF	shoot fresh weight (g)	shoot dry weight (g)	root fresh weight (g)	root dry weight (g)	total biomass (g)
treatment	4	76.91^***^	128.01^***^	495.5^***^	479.54^***^	659.5^***^
variety	1	496.04^***^	647.31^***^	1677.36^***^	1577.61^***^	3855.8^***^
treatment × variety	4	74.81^***^	35.11^***^	341.31^***^	342.47^***^	583.2^***^
error	25	0.86	0.84	1.13	1.17	9.5

source	DF	leaf temperature	photosynthetic rate	transpiration rate	stomatal conductance	WUE
treatment	4	1.077^***^	0.37646^***^	0.37646^***^	356.52^***^	1182.26^***^
variety	1	105.235^***^	0.34812^***^	0.34812^***^	0.26^***^	2206.95^***^
treatment × variety	4	2.575^***^	0.30205^***^	0.30205^***^	246.13^***^	1023.05^***^
error	25	0.138	0.00547	0.00547	0.1	3.47

source	DF	shoot fresh weight (g)	shoot dry weight (g)	root fresh weight (g)	root dry weight (g)	total biomass (g)
treatment	4	76.91^***^	128.01^***^	495.5^***^	479.54^***^	659.5^***^
variety	1	496.04^***^	647.31^***^	1677.36^***^	1577.61^***^	3855.8^***^
treatment × variety	4	74.81^***^	35.11^***^	341.31^***^	342.47^***^	583.2^***^
error	25	0.86	0.84	1.13	1.17	9.5

source	DF	leaf temperature	photosynthetic rate	transpiration rate	stomatal conductance	WUE
treatment	4	1.077^***^	0.37646^***^	0.37646^***^	356.52^***^	1182.26^***^
variety	1	105.235^***^	0.34812^***^	0.34812^***^	0.26^***^	2206.95^***^
treatment × variety	4	2.575^***^	0.30205^***^	0.30205^***^	246.13^***^	1023.05^***^
error	25	0.138	0.00547	0.00547	0.1	3.47

source	DF	cob length (cm)	cob diameter (cm)	cob weight (g)	biological yield (g)	grain yield (g)	harvest index (%)
treatment	4	377.7^***^	162.66^***^	0.093^***^	230.64^***^	2.53^***^	0.024*
variety	1	39.35^***^	86.87^***^	6.034^***^	683.10^***^	5.27*	0.024*
treatment × variety	4	174.35^***^	539.53^***^	0.97^***^	1324.28^***^	16.29^**^	0.014^**^
error	25	9.63	1.96	0.027	1.83	0.05	0.002

source	DF	leaf temperature	photosynthetic rate	transpiration rate	stomatal conductance	WUE
treatment	4	1.077^***^	0.37646^***^	0.37646^***^	356.52^***^	1182.26^***^
variety	1	105.235^***^	0.34812^***^	0.34812^***^	0.26^***^	2206.95^***^
treatment × variety	4	2.575^***^	0.30205^***^	0.30205^***^	246.13^***^	1023.05^***^
error	25	0.138	0.00547	0.00547	0.1	3.47

source	DF	cob length (cm)	cob diameter (cm)	cob weight (g)	biological yield (g)	grain yield (g)	harvest index (%)
treatment	4	377.7^***^	162.66^***^	0.093^***^	230.64^***^	2.53^***^	0.024*
variety	1	39.35^***^	86.87^***^	6.034^***^	683.10^***^	5.27*	0.024*
treatment × variety	4	174.35^***^	539.53^***^	0.97^***^	1324.28^***^	16.29^**^	0.014^**^
error	25	9.63	1.96	0.027	1.83	0.05	0.002

#### 3.2.3 Transpiration Rate (μg H_2_O m^–2^ S^–1^)

ANOVA presented in Table 6 depicts the significant variation between maize varieties and PM treatments and their combined interaction of varieties on the transpiration rate. A significant decrease in the transpiration rate of Pearl (ranging from 7.5 to 77.8%) and an increase in the rate of MMRI (ranging from 0.0 to 95.6%) was observed as compared to their respective control. In Pearl (V_1_), minimum decrease (0.7 μg H_2_O m^–2^ S^–1^) in transpiration rate was obtained at 75 g/pot of PM and maximum (0.2 μg H_2_O m^–2^ S^–1^) at 50 g/pot of PM, whereas in the case of MMRI (V_2_), minimum increase (0.4 μg H_2_O m^–2^ S^–1^) was observed at 25 g/pot of PM and maximum (1.1 μg H_2_O m^–2^ S^–1^) at 50 g/pot of PM in the soil as compared to the control (Figure 3 and Table 5).

#### 3.2.4 Stomatal Conductance (mmol m^–2^ s^–1^)

ANOVA presented in Table 6 depicts the significant variation between maize varieties and PM treatments and their combined interaction of varieties on stomatal conductance. A significant increase in stomatal conductance of Pearl (ranging from 54.25 to 71.23%) with minimum increase (25.4 mmol m^–2^ s^–1^) at 50 g/pot of PM and maximum (28.2 mmol m^–2^ s^–1^) at 25 g/pot of PM in the soil. Stomatal conductance was increased in MMRI up to 100 and 11% at 50 and 75 g/pot, while it was decreased at 25 and 100 g/pot of PM in the soil as compared to the control (Figure 3 and Table 5).

#### 3.2.5 WUE (%)

ANOVA presented in Table 6 depicts the significant variation between maize varieties and PM treatments and their combined interaction of varieties on the WUE. A significant increase in the WUE of Pearl (ranging from 16.45 to 99.42%) and MMRI (ranging from 38.47 to 70.07%) was observed as compared to their respective control. In Pearl (V_1_), minimum increase (35.6%) in WUE was obtained at 25 g/pot of PM and maximum (61.6%) at 100 g/pot of PM, whereas in the case of MMRI (V_2_), minimum increase (74.6%) was observed at 75 g/pot of PM and maximum (91.6%) at 25 g/pot of PM in the soil as compared to the control (Figure 3 and Table 5).

### 3.3 Yield and Yield-Related Traits

#### 3.3.1 Cob Length (cm)

ANOVA presented in Table 6 depicts the significant variation between maize varieties and PM treatments and their combined interaction of varieties on the cob length. A significant increase in the cob length of Pearl (ranging from 25.84 to 46.09%) and MMRI (ranging from 27.74 to 47.44%) was observed as compared to their respective control. In Pearl (V_1_), minimum cob length (17.54 cm) was measured at 75 g/pot of PM and maximum (20.36 cm) at 100 g/pot of PM, whereas in the case of MMRI (V_2_), minimum cob length (27.74 cm) was observed at 25 g/pot of PM, and maximum (47.44 cm) at 100 g/pot of PM in the soil as compared to the control (Table 6).

#### 3.3.2 Cob Diameter (cm)

ANOVA presented in Table 6 depicts the significant variation between maize varieties and PM treatments and their combined interaction of varieties on the cob diameter. A significant increase in the cob diameter of Pearl (ranging from 7.18 to 15.75%) and MMRI (ranging from 6.13 to 8.53%) was observed as compared to their respective control. In Pearl (V_1_), minimum cob diameter (3.88 cm) was obtained at 75 g/pot of PM and maximum (4.19 cm) at 100 g/pot of PM, whereas in the case of MMRI (V_2_), minimum cob diameter (3.93 cm) was observed at 100 g/pot of PM, and maximum (4.07 cm) at 75 g/pot of PM in the soil as compared to the control.

#### 3.3.3 Cob Weight (g)

ANOVA presented in Table 6 depicts the significant variation between maize varieties and PM treatments and their combined interaction of varieties on the cob weight. A significant increase in the cob weight of Pearl (ranging from 34.87 to 69.06%) and MMRI (ranging from 36.95 to 54.52%) was observed as compared to their respective control. In Pearl (V_1_), minimum cob weight (68.06 g) was obtained at 75 g/pot of PM and maximum (85.31 g) at 100 g/pot of PM. In the case of MMRI (V_2_), minimum cob weight (77.18 g) was observed at 25 g/pot of PM, and maximum cob weight (87.09 g) was measured at 100 g/pot of PM in the soil as compared to the control (Table 6).

#### 3.3.4 Biological Yield (g)

ANOVA presented in Table 6 depicts the significant variation between maize varieties and PM treatments and their combined interaction of varieties on the biological yield. A significant increase in the biological yield of Pearl (ranging from 4.40 to 8.36%) and MMRI (ranging from 1.15 to 3.52%) was observed as compared to their respective control. In Pearl (V_1_), minimum biological yield (19.24 g) was obtained at 25 g/pot of PM and maximum (19.97 g) at 100 g/pot of PM. In the case of MMRI (V_2_), minimum biological yield (19.28 g) was observed at 25 g/pot of PM, and maximum biological yield (19.73 g) was measured at 100 g/pot of PM in the soil as compared to the control (Tables 6 and 7).

**Table 7:** Yield-Related Traits of Maize

treatments	V_1_ V_2_		V_1_ V_2_		V_1_ V_2_		V_1_ V_2_		V1 V2		V1 V2	
	biological yield		grain yield		harvest index		crude protein		ash content		fiber content	
T_0_	18.43	19.06	5.31	5.69	28.81	29.85	4.79	5.43	2.36	2.09	1.79	2.31
T_1_	19.24	19.37	6.04	6.58	31.39	34.13	3.26	3.34	6.57	2.10	5.08	2.71
T_2_	19.75	19.59	6.09	6.69	30.84	34.54	3.42	3.18	5.52	2.31	4.62	3.04
T_3_	19.84	19.73	6.13	6.83	30.90	34.86	3.66	3.40	3.94	5.68	3.93	4.65
T_4_	19.97	19.28	6.17	6.95	30.90	35.23	3.67	3.93	4.32	5.87	4.13	4.75

#### 3.3.5 Grain Yield (g)

ANOVA presented in Table 6 depicts the significant variation between maize varieties and PM treatments and their combined interaction of varieties on the grain yield. A significant increase in the grain yield of Pearl (ranging from 13.75 to 16.20%) and MMRI (ranging from 15.64 to 22.14%) was observed as compared to their respective control. In Pearl (V_1_), minimum grain yield (6.04 g) was obtained at 25 g/pot of PM and maximum (6.17 g) at 100 g/pot of PM. In the case of MMRI (V_2_), minimum grain yield (6.58 g) was observed at 25 g/pot of PM, and maximum grain yield (6.95 g) was measured at 100 g/pot of PM in the soil as compared to the control (Tables 6 and 7).

#### 3.3.6 Harvest Index (%)

ANOVA presented in Table 6 depicts the significant variation between maize varieties and PM treatments and their combined interaction of varieties on the harvest index. A significant increase in the harvest index of Pearl (ranging from 7.03 to 8.97%) and MMRI (ranging from 14.33 to 18.01%) was observed as compared to their respective control. In Pearl (V_1_), minimum harvest index (30.84%) was obtained at 50 g/pot of PM and maximum (31.39%) at 25 g/pot of PM. In the case of MMRI (V_2_), minimum harvest index (34.13%) was observed at 25 g/pot of PM, and maximum harvest index (35.23%) was measured at 100 g/pot of PM in the soil as compared to the control (Tables 6 and 7).

#### 3.3.7 Crude Protein (mg/g)

**Figure 4:**
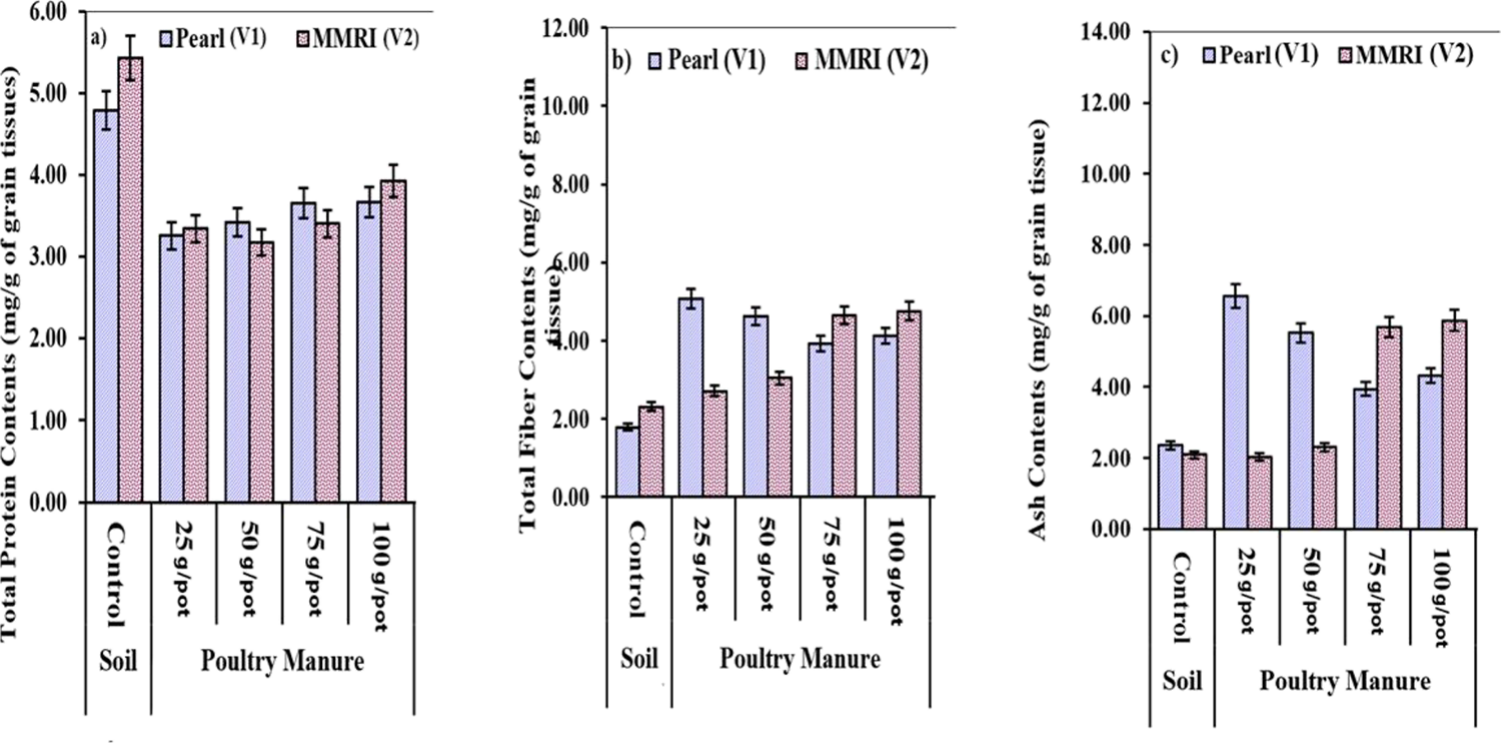
Effect of PM on the protein content, fiber content, and ash content.

Table 7 shows the effect of PM on the yield of maize verities. In Pearl (V_1_), minimum crude protein (3.26 mg/g) was obtained at 75 g/pot of PM and maximum (4.79 mg/g) in the control, whereas in the case of MMRI (V_2_), minimum (3.18 mg/g) was observed at 50 g/pot of PM and maximum (5.43 mg/g) in the control (Figure 4).

#### 3.3.8 Ash Content (mg/g)

Table 7 shows the effect of PM on the yield of maize verities. In Pearl (V_1_), minimum ash content (2.36 mg/g) was obtained in the control and maximum (6.57 mg/g) in T_1_, whereas in the case of MMRI (V_2_), minimum (2.09 mg/g) was observed in the control and maximum (5.87 mg/g) in T_4_ (Figure 4).

#### 3.3.9 Fiber Content (mg/g)

Table 7 shows the effect of PM on the yield of maize verities. In Pearl (V_1_), minimum fiber content (1.79 mg/g) was obtained in the control and maximum (5.08 mg/g) in T_1_, whereas in the case of MMRI (V_2_), minimum (2.31 mg/g) was observed in the control and maximum (4.75 mg/g) in T_4_ (Figure 4).

##### 3.3.9.1 Correlation

**Figure 5:**
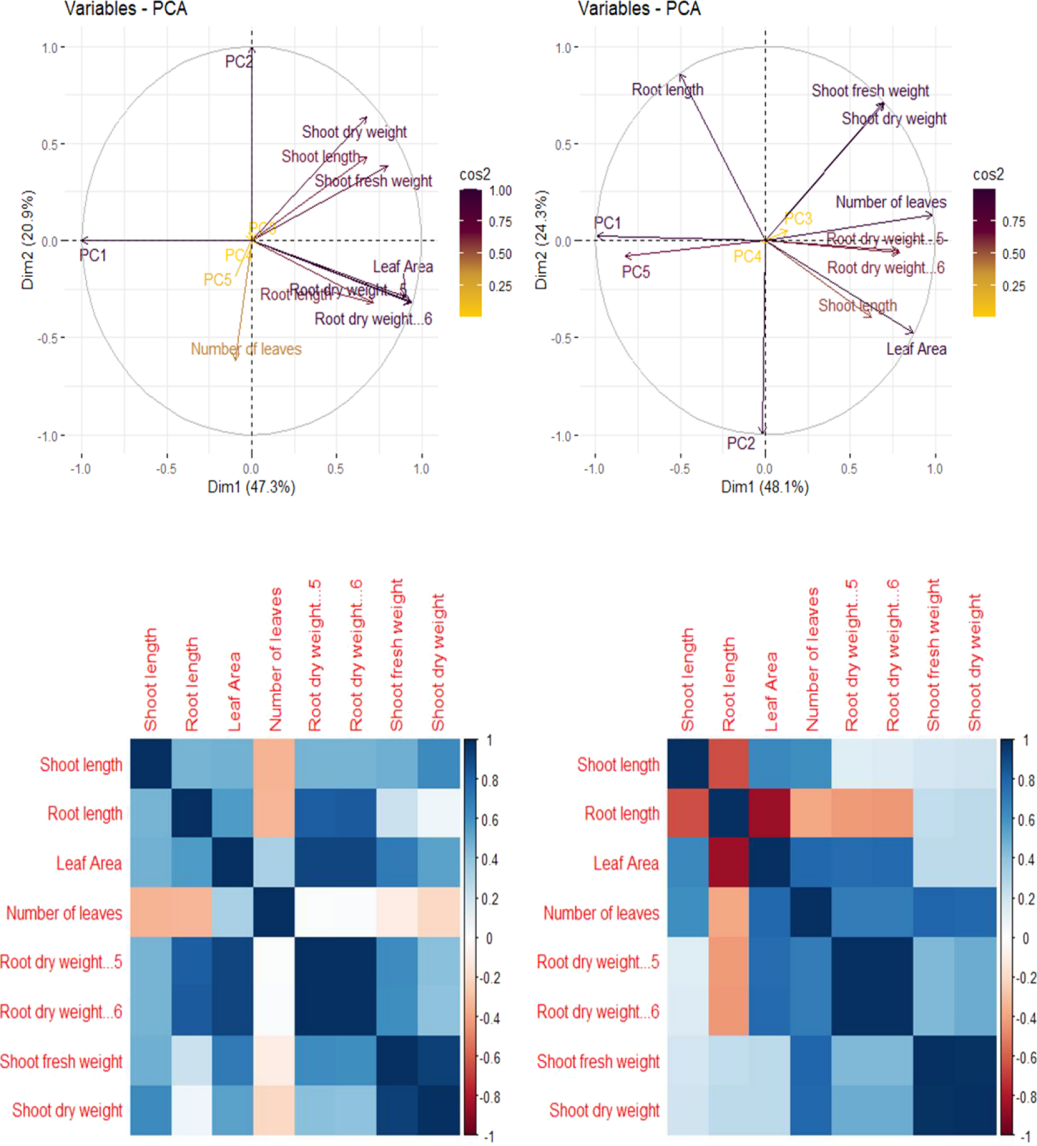
PCA (a) and Pearson correlation (b) showing association among the different variables of the maize plant.

Principal component analysis (PCA) and correlation presented in (Figure 5a,b) show the correlation among different growth parameters of the maize plant. In PCA, in the VI variety, Dim1 (PCA-1) comprised 47.3% and Dim2 (PCA-2) comprised 20.9%, while in V2, Dim1 (PCA-1) comprised 48.1% and Dim2 (PCA-2) comprised 24.3% of the whole database. In both types of soil, all variables dispersed successfully in the whole database, and it was noticed that the shoot fresh weight, the shoot length, and the shoot dry weight are positively correlated, while the root dry weight and the leaf area are negatively correlated in V1. The shoot fresh weight, the shoot dry weight, and the number of leaves are positively correlated, while the root length, the root dry weight, and the shoot length are negatively correlated in V2.

## 4 Discussion

Various agricultural practices are needed to increase the maize growth, the grain yield and yield components, and the nutritional quality of maize grains as the soil of arable land areas has very low organic content and nutrient deficient.^28−32^ Therefore, most of the crops show positive response to soil amendments with various products.^33−35^ PM is considered as a rich source of nutrients as it improves soil fertility and crop production, and it is an enriched source of various major macronutrients such as nitrogen (N), potassium (K), and phosphorus (P).^36,37^ Important plant nutrients like nitrogen, phosphorous, potassium, calcium, magnesium, sulfur, copper, zinc, chlorine, boron, iron, and molybdenum, all are present in PM. So, PM can be utilized as a good fertilizer to fulfill all or a portion of nutritional requirements of crop plants. Moreover, PM has good carbon-to-nitrogen ratio which facilitates the microorganisms that ultimately improves the soil properties and plant productivity.^38,39^ Addition of PM increases the cation-exchange capacity of the soil.^40^ Therefore, the present study was designed to investigate the effects of soil amendments with different levels (25, 50, 75, and 100 g/pot) of PM on the growth, physiology, and yield of two maize varieties, that is, Pearl and MMRI.

Plant height, root length, fresh and dry weight of shoot, fresh and dry weight of root, number of leaves, leaf area, and total biomass production were significantly increased in both maize varieties, that is, Pearl (31.0, 63.2, 12.5, 43.9, 95.6, 99.3, 30.8, 78.1, and 72.2%, respectively) and MMRI (46.9, 2.2, 57.3, 79.6, 29.6, 78.4, 46.7, 91.8, and 62.4%, respectively), grown in soil amended with different levels of PM as compared to their respective control. Seedling growth response of both maize varieties was different in each treatment of PM, but an increase in the overall average values of all morphological traits was recorded at 75 g/pot of PM addition to the soil. Previous studies also confirm that PM and excreta of other animals are appropriate to enhance the seedling growth at different stages.^41,42^ Boateng et al.^43^ and Farhad et al.^44^ had also reported that PM improves the soil fertility by providing various essential nutrients such as nitrogen, phosphorus, and potassium and organic contents and also maintains the level of exchangeable cations.

Physiological attributes such as the leaf area index, the leaf temperature, the transpiration rate, the photosynthetic rate, the WUE, and stomatal conductance play a significant role in plant growth and production.^45−49^ These physiological attributes are sensitive to growth conditions such as nutrients and water availability in the soil and various other environmental factors, that is, temperature, drought,^50−54^ salinity,^55^ and heavy metal.^56−63^ Leaf area index, leaf temperature, transpiration rate, photosynthetic rate, WUE, and stomatal conductance were increased in both maize varieties, Pearl (77.8, 3.24, 2.10, 77.8, 9.60, 92.42, and 71.23%, respectively) and MMRI (91.2, 0.17, 3.96, 95.6, 4.5, 53.62, 70.07, and 100%, respectively), grown in soil amended with different levels of PM as compared to their respective control. Both maize varieties differently responded to each treatment of PM for each physiological trait. The increase over control in all physiological traits under study in response to PM was more significant as compared to the control. Agronomic and yield traits of both maize varieties were compared to check the effects of soil amendments with different levels of PM (25, 50, 75, and 100 g/pot). Cob length, cob diameter, cob weight, number of grains per cob, grain yield, and biological yield was significantly increased in both maize varieties i.e., Pearl (46.09, 15.75, 69.06, 11.47, 16.20, and 8.36%, respectively) and MMRI (47.44, 8.53, 54.52, 13.26, 22.14, and 3.52%, respectively) grown in soil amended with 100 g/pot of PM as compared to their respective control. Increase in the economic yield and its components in maize varieties due to the addition of PM manure in the soil was also reported.^43,44^ Fulhage^41^ and Bargali^42^ reported that PM is an enriched source of macronutrients such as nitrogen (N), potassium (K), and phosphorus (P), which improve soil fertility and crop production.

## 5 Conclusions

In conclusion, the results of this study suggest that the use of PM as a soil amendment has a positive impact on the growth and yield of maize. The addition of PM to the soil improved soil physical and chemical properties such as available P and soil organic matter. Furthermore, the growth and physiological attributes of both maize varieties were significantly enhanced in the soil amended with PM compared to their respective control. Present study revealed that the soil amended with 25 g/pot of PM was the most effective byproduct for maize shoot biomass production and maize harvest index in Pearl (V_1_). 50 g/pot PM was the most effective byproduct for root growth, leaf area, leaf area index, and total biomass production of maize cultivars in Pearl (V_1_). It is concluded from the present study that the soil amended with 75 g/pot of PM enhanced the plant height in grains of both maize varieties. Relative humidity of MMRI (V_2_) was enhanced by soil amended with 50 g/pot of PM. Soil amended with 100 g/pot of PM significantly enhanced the photosynthetic rate, transpiration rate, WUE, cob length, cob diameter, cob weight, number of grains per cob, grain yield, and biological yield in MMRI (V_2_). It is concluded from the present study that from the overall comparison of varieties, variety V_2_ (MMRI) showed better results as compared to V_1_ (Pearl) with respect to all parameters, that is, plant height, leaf temperature, relative humidity, transpiration rate, photosynthetic rate, leaf area, WUE, stomata conductance, cob length, cob diameter, cob weight, number of grains per cob, grain yield, biological yield, and number of leaves. PM improved the overall growth in both varieties. Generally, it can be concluded from the current research work that V_2_ (MMRI) showed better results with respect to most parameters, and PM proved of greater quality for the overall growth, yield, physiological, and biochemical attributes. This research provides an eco-friendly and economically effective method for the safe disposal of industrial byproducts while simultaneously addressing the issue of environmental pollution. Overall, the findings of this study can reform agricultural practices aimed at improving soil quality and increasing crop productivity in a sustainable manner.
